# Novel therapeutic and diagnostic antibodies against KIR3DL2, a unique tumor antigen overexpressed on subtypes of T cell lymphomas

**DOI:** 10.1186/2051-1426-1-S1-P45

**Published:** 2013-11-07

**Authors:** Anne Marie-Cardine, Nicolas Viaud, Arnaud Dujardin, Joly Rachel, Nathalie Granier, Laurent Gauthier, Cecile Bonnafous, Mathieu Blery, Carine Paturel, Armand Bensussan, Martine Bagot, Helene Sicard

**Affiliations:** 1 R&D, Innate Pharma, Marseille, France; 2 INSERM U976, Hopital St Louis, Paris, France

## 

KIR3DL2 belongs to the killer immunoglobulin (Ig)-like receptors (KIRs) family and is composed of three extracellular Ig-like domains. KIR3DL2 is naturally expressed on some NK cells and minor subpopulations of CD8+ and CD4+ T cells. Physiologically, KIRD3L2 is an inhibitory receptor for human leukocyte antigen (HLA) class I molecules regulating NK cell activation. Remarkably, KIR3DL2 is also aberrantly overexpressed on several subtypes of T lymphomas/leukemias, such as Sezary Syndrome, transformed Mycosis Fungoides and HTLV1+ Adult T Cell Leukemia, making it a unique therapeutic target in cancer. We have generated a series of anti-KIR3DL2 monoclonal antibodies (mAbs) binding selectively to KIR3DL2, spanning epitopes on all three Ig domains. Their efficacy was evaluated in vitro and in vivo against KIR3DL2-expressing tumors and Sezary cell lines as disease model. An autologous assay was set up to evaluate the killing of primary Sezary cells by patients’ NK effectors in the presence of those mAbs. Potent antibody-dependent cell cytotoxicity (ADCC) was found the main mode of action involved in their anti-tumor activity. The most promising candidates were humanized as IgG1 mAbs and the final lead molecule was selected for further development, based on several predefined criteria. In parallel, anti-KIR3DL2 mAbs were also developed as unique and sensitive tools for the detection by immunohistochemistry of KIR3DL2 on tumor biopsies. Owing to the promising efficacy profile of our anti-KIR3DL2 mAb candidate and to the highly restricted expression pattern of the target on some T leukemia/lymphoma cells, an antibody-based therapy targeting KIR3DL2 stands as a potentially unequalled strategy in several orphan diseases with critical unmet medical need.

